# Investigation Outcomes of Tuberculosis Suspects in the Health Centers of Addis Ababa, Ethiopia

**DOI:** 10.1371/journal.pone.0018614

**Published:** 2011-04-19

**Authors:** Amare Deribew, Nebiyu Negussu, Zenebe Melaku, Kebede Deribe

**Affiliations:** 1 Department of Epidemiology, Jimma University, Jimma, Ethiopia; 2 Somali Regional Health Bureau, Jijiga, Ethiopia; 3 International Center for AIDS Care and Treatment Program (ICAP), Addis Ababa, Ethiopia; 4 Department of General Public Health, Jimma University, Jimma, Ethiopia; Dirección General de Epidemiología, Peru

## Abstract

**Background:**

Little is known about the prevalence of tuberculosis (TB) and HIV among TB suspects in primary health care units in Ethiopia.

**Methods:**

In the period of February to March, 2009, a cross sectional survey was done in 27 health centers of Addis Ababa to assess the prevalence of TB and HIV among TB suspects who have > = 2 weeks symptoms of TB such as cough, fever and weight loss. Diagnosis of TB and HIV was based on the national guidelines. Information concerning socio-demographic variables and knowledge of the respondents about TB was collected using pretested questionnaire.

**Results:**

Of the 545 TB suspects, 506 (92.7%) of them participated in the study. The prevalence of both pulmonary and extra pulmonary TB was 46.0% (233/506). The smear positivity rate among pulmonary TB suspect was 21.3%. Of the TB suspects, 298 (58.9%) of them were tested for HIV and 27.2% (81/298) were HIV seropositive. Fifty percent of the HIV positive TB suspects had TB. TB suspects who had a contact history with a TB patient in the family were 9 times more likely to have TB than those who did not have a contact history, [OR = 9.1, (95%CI:4.0, 20.5)]. Individuals who had poor [OR = 5.2, (95%CI: 2.3, 11.2)] and fair knowledge [OR = 3.7, (95%CI: 1.3, 10.4)] about TB were more likely to have TB than individuals who had good knowledge.

**Conclusion:**

In conclusion, the prevalence of TB among TB suspects with duration of 2 or more weeks is high. Fifty percent of the HIV positive TB suspects had TB. Case finding among TB suspects with duration of 2 or more weeks should be intensified particularly among those who have a contact history with a TB patient.

## Introduction

One third of the global population is infected by *mycobacterium tuberculosis*. Eighty percent of the total new tuberculosis (TB) in the world are found in 22 high burden countries[1]. Since the last 50 years, TB has been recognized as a major public health problem of Ethiopia[2]. The World Health Organization (WHO) estimated that Ethiopia is the seventh high burden country in terms of incidence cases of TB[1].

Low case detection rate (34%) of smear positive TB[2], high burden of TB/HIV co-infection, ranging from 25% to 57% [3], [4], [5], [6], [7] and the emergence of multi-drug resistance TB pose serious challenges for the TB control program in Ethiopia[1], [2]. The country has been implementing the TB/HIV collaborative activities such as screening of TB patients for HIV, intensified TB case finding among HIV patients and joint coordination of the two diseases since 2007[8].

Screening of TB suspects using symptom based questionnaires provides a quick, cheap and convenient way to identify individuals at a high risk of tuberculosis (TB). Individuals identified as TB suspects by the simple clinical algorithm need to be investigated further using definitive tests such as sputum microscopy, chest radiography and, TB culture if it is available. More recently, provider-initiated TB screening (i.e. active case-finding) has become an important part of HIV care in resource-poor settings including Ethiopia [9]–[14]. Ruling out active TB is necessary both for individual patient management and for TB infection control. In patients starting antiretroviral therapy, undiagnosed TB is common and carries a substantially increased risk of death or hospitalization in the first few months of treatment [14], [15].

In Ethiopia, little is known about the prevalence of TB, HIV and TB/HIV co-infection among TB suspects with duration of cough of two or more weeks in the primary health care units (health centers). The objective of this study was to evaluate the prevalence of TB and HIV among TB suspects in Addis Ababa using simple symptom based questionnaire. Participants who were identified as TB suspects were further investigated for TB and HIV using sputum analysis and rapid HIV testing.

## Methods

### Setting and period

The study was conducted from February to March, 2009 in twenty-seven public health centers (primary health care units) in Addis Ababa, the capital city of the Federal democratic Republic of Ethiopia. These health centers provide curative and preventive health services for the community of Addis Ababa which has an estimated total population of 2,917,295[16].

### Study design

A facility based cross-sectional study was conducted in 27 health centers in Addis Ababa.

### Study population and sampling

Study participants were 545 adult pulmonary TB or extrapulmonary TB (TB lymphadenitis) suspects (> = 15 years) identified in the study health centers. Other forms of extrapulmonary TB suspects were excluded from the study. Considering resources, the sample size was estimated using a single proportion sample size formulae by considering the following parameters: prevalence of TB among adult pulmonary TB suspects of 29% [6], 95% CI, and 4% of margin of error and 10% for the non-response rate.

### Measurement

TB suspects were identified using a pre-tested questionnaire. TB suspects were defined as individuals who had cough of ≥2 weeks OR two or more of the following symptoms: *weight loss, fever, excessive night sweats and painless swelling of cervical or axillary lymph nodes* of more than 2 weeks. Diagnosis of TB among TB suspects was based on the national TB guideline[2]. Three consecutive sputum samples (spot-morning-spot) were collected and examined for the presence of Acid Fast Bacilli (AFB) using the standard Ziehl-Neelsen method[17]. As a quality control method, the entire positive and 10% of the negative slides were re-read by experienced laboratory technicians. Pulmonary TB suspects who had two smear positive results were categorized as smear positive. TB suspects who had three negative smears and suggestive X-ray findings or failure to respond to an antibiotic trials were categorized as smear negative pulmonary TB. TB lymphadenitis was diagnosed based on clinical parameters and cytological examination obtained by fine needle aspiration. Diagnosis of HIV was based on national guideline and the detail procedure is published elsewhere[18].

Information concerning the socio-demographic and knowledge of patients on TB was collected by trained general practitioners or nurses using a pretested and previously used questionnaire [19]. The questionnaire was originally developed in English and translated to Amharic (local language). To ensure consistency, the questionnaire was retranslated to English by another person who was blind to the original questionnaire. There were 24 knowledge questions about TB. The questions focused on sign and symptoms, preventive methods, treatment of TB. For each question 0 is given for incorrect answers and 1 for correct answers. A person was considered to have good, fair or poor knowledge if he/she answered respectively more than 75%, 50–75%, or less than 50% of the questions correctly.

### Data analysis

Data were entered into SPSS Version 16.0 statistical software. Univariate analysis was done to describe the socio-demographic characteristics of the study participants and prevalence of TB. A bivariate analysis using binary logistic regression was done to determine the presence of a statistically significant association between explanatory variables and the outcome variables (presence of TB or HIV). To identify independently associated factors, multiple logistic regression model was produced by taking presence of TB as outcome variable. All explanatory variables that were associated with the outcome variable in the bivariate analysis (P = ≤0.2) and variables consistently found to be associated with occurrence of TB in other studies were included in the logistic regression model. Odds Ratio (OR) and their 95% Confidence Intervals (CI) were calculated.

### Ethical consideration

Ethical clearance was obtained from the ethical committee of Jimma University and the Addis Ababa health bureau. Written consent was obtained from each participants and confidentiality was assured for all the information provided. Identification of a participant was only through numerical codes.

## Results

### Prevalence of TB among TB suspects

Of the total TB suspects during the study period (n = 545), 506(92.7%) of them participated in the study. The prevalence of both pulmonary and extra pulmonary TB was 46.0% (233/506). Of the TB patients, 100(42.9%), 96(41.2%) and 37(15.9) had smear positive, smear negative and extra pulmonary TB. The smear positivity rate among pulmonary TB suspect was 21.3% (100/469).

There was no difference in sex, age, literacy status, occupation and marital status between patients with and without evidence of TB (**Table-1**).

**Table 1:** Association of socio-demographic characteristics and TB Addis Ababa, March 2009.

Socio-demographic variables	Presence of TB Disease		P-value
	Yes, no (%)	No, n (%)	
**Sex**			0.89
Males	113(48.5)	134(49.1)	
Females	120(51.5)	139(50.9)	
**Age in years**			0.59
15–24	64(27.5)	63(23.1)	
25–34	67(28.8)	78(28.6)	
35–49	68(29.2)	83(30.4)	
> = 50	34(14.6)	49(17.9)	
**Educational status**			0.57
Illiterate	67(28.8)	96(35.2)	
Can read and write	95(40.8)	99(36.3)	
Elementary	22(9.4)	21(7.7)	
High school	38(16.3)	46(16.8)	
Above high school	11(4.7)	11(4.0)	
**Occupation**			0.89
House wives	56(24.0)	65(23.8)	
Merchant	32(13.7)	44(16.1)	
Government employee	30(12.9)	32(11.7)	
Day laborers	97(41.6)	107(39.2)	
Jobless	18(7.7)	25(9.2)	
**Marital status**			0.22
Single	104(44.6)	102(37.4)	
Married	97(41.6)	124(45.4)	
Divorced	32(13.7)	47(17.2)	

(n = 506).

### HIV prevalence among TB suspects

Of the TB suspects, 298(58.9%) of them were tested for HIV. The prevalence of HIV was 27.2% (81/298). The HIV positive and negative individuals had no significant differences concerning sex, educational status, and marital status. Larger proportion of housewives and merchants were HIV positive (P = 0.025). The prevalence of HIV was higher in the age group of 25–34 and > = 50 years (P = 0.014). Fifty percent of the HIV positive (41/81) and 48.4%(105/217) of the HIV negative TB suspects had TB (P = 0.32) (**Table-2**).

**Table 2:** Association of socio-demographic characteristics and HIV serostatus Addis Ababa, March 2009.

Socio-demographic variables	HIV serostatus		P-value
	Positive, no (%)	Negative, no (%)	
**Sex**			0.71
Males	40(49.4)	102(47.0)	
Females	41(50.6)	115(53.0)	
**Age in years**			0.014
15–24	10(12.3)	61(28.1)	
25–34	32(39.5)	62(28.6)	
35–49	23(28.4)	67(30.9)	
> = 50	16(19.8)	27(12.4)	
**Educational status**			0.84
Illiterate	26(32.1)	61(28.1)	
Can read and write	34(42.0)	87(40.1)	
Elementary	6(7.4)	23(10.6)	
High school	12(14.8)	34(15.7)	
Above high school	3(3.7)	12(5.5)	
**Occupation**			0.025
House wives	21(25.9)	49(22.6)	
Merchant	15(18.5)	38(17.5)	
Government employee	12(14.8)	32(14.7)	
Day laborers	22(27.2)	89(41.0)	
Jobless	11(13.6)	9(4.1)	
**Marital status**			0.35
Single	29(35.8)	93(42.9)	
Married	36(44.4)	94(43.3)	
Divorced	16(19.8)	30(13.8)	
**Infection with TB**			0.32
No TB	40(49.4)	112(51.6)	
Pulmonary TB	31(38.3)	90(41.5)	
Extra pulmonary TB	10(12.3)	15(6.9)	
Both PTB and ETB	40(49.4)	102(47.0)	

(n = 298).

### The association between symptoms and occurrence of pulmonary TB

There was no statistically significant association between chronic cough, night sweating, loss of appetite, weight loss, and fever with the occurrence of pulmonary TB. Those who had chest pain were 1.5 times more likely to have pulmonary TB than those who did not have chest pain, [OR = 1.5, (95%CI: 1.0, 2.28)] (**Table-3**).

**Table 3:** Association of symptoms with the occurrence of pulmonary TB, Addis Ababa, March 2009.

Variables	Presence of TB disease		Crude OR(95%CI)	Adjusted OR(95% CI)
	Yes, no (%)	No, no (%)		
**Cough**				
Yes	181(92.3)	245(89.7)	1.37(0.72,2.65)	-
No	15(7.7)	28(10.3)	1	
**Night sweating**				
Yes	84(42.9)	130(47.6)	0.82 (0.57.1.19)	-
No	112(57.1)	143(52.4)	1	
**Loss of appetite**				
Yes	40(20.4)	70(25.6)	0.74(0.47,1.15)	-
No	156(79.6)	203(74.4)	1	
**Weight loss**				
Yes	37(18.9)	63(23.1)	0.77(0.49,1.22)	-
No	159(81.1)	21(7.7)	1	
**Fever**				
Yes	33(16.8)	41(15.0)	1.20(0.69,1.88)	-
No	163(83.2)	232(85.0)	1	
**Shortness of breath**				
Yes	10(5.1)	7(2.6)	2.0(0.76,5.46)	-
No	186(94.9)	266(97.4)	1	
**Chest pain**				
Yes	63(32.1)	65(23.8)	1.50(1.0,2.28)	-
No	133(67.9)	208(76.2)	1	
**Contact with TB patient**				
No	138(70.4)	256(93.8)	1	1
Yes	58(29.6)	17(6.2)	5.2(2.9,9.2)	9.1(4.0,20.5)
**Knowledge about TB**				
Good	16(8.2)	84(30.8)	1	1
Fair	23(11.7)	30(11.0)	4.0(1.8,8.6)	3.7(1.3,10.4)
Poor	157(80.1)	159(58.2)	6.4 (3.6,11.4)	5.2(2.3,11.2)
**HIV status**				
Negative	90(74.4)	112(73.7)	1	1
Positive	31(25.6)	40(26.3)	1.1(0.6,1.8)	0.8(0.4,1.5)
**Number of people per room in a house(average = 2.3 persons/room)**				
Below average	110(61.7)	168(61.5)	1	1
Above average	86(56.1)	105(38.5)	1.1(0.8,1.6)	1.3(0.7,2.2)
**Smoking**				
No	171(87.2)	241(88.3)	1	1
Yes	25(12.8)	32(11.7)	1.1(0.6,1.8)	1.2(0.5,2.7)

(n = 469).

### Factors associated with pulmonary TB

TB suspects who had a contact history with a TB patient in the family were 9 times more likely to have TB than those who did not have a contact history, [OR = 9.1,(4.0, 20.5)]. Individuals who had poor [OR = 5.2, (95%CI: 2.3, 11.2)] and fair knowledge [OR = 3.7, (95%CI: 1.3, 10.4)] about TB were more likely to have TB than individuals who had good knowledge. Number of people per room in a household, and smoking did not have association with the occurrence of TB (**Table-3**).

## Discussion

In this study several important findings were observed. The prevalence of both pulmonary and extra pulmonary TB among suspects was 46.0%. The smear positivity rate among pulmonary TB suspect was 21.3%. Of the TB suspects, 27.2% were found to be HIV positive. Half of the HIV positive TB suspects had TB. Low knowledge of TB and contact history were predictors of active TB among suspects. None of the single TB symptoms were associated with occurrence of pulmonary TB.

The smear positivity rate in our study (21.3%) was higher than previous reports in Mali (10%)[20], Ethiopia (15.6%)[21] and Mexico (7.5%) [22], however a high (47.8%) prevalence was observed among patients with cough of more than 3 weeks in Latin America [23]. The prevalence of pulmonary TB in this study is also higher than the finding of Bruchfeld et al who identified a prevalence of 19% using microscopy among TB suspects who had cough of more than 3 weeks in Ethiopia[6]. The prevalence of TB among suspects in the health institution is also higher than the prevalence in the general population of urban[24], [25] and rural[26] Ethiopia. The prevalence of HIV among TB suspects (27%) was comparable with the finding of Yassin et al in south Ethiopia[7], but lower than the reports of Odhiambo et al in Kenya (61%)[27], and Srikantiah et al in Uganda (42%)[28]. The difference could be due to the difference in the general HIV prevalence in the countries. The proportion of extra pulmonary TB(15.9%) in this study is lower compared to the national estimate (34%)[2]. The difference could be due to over diagnosis of extrapulmonary TB in rural health centers in Ethiopia where there is no laboratory facilities.

In this study, there was no statistically significant association between HIV and TB disease. Results on the association between HIV and TB among TB suspects are conflicting. A study in Kenya revealed no association between HIV and TB[27]. On the other hand, a study in Uganda[28] showed that HIV prevalence was higher among non-TB patients than TB patients, Brushfeld et al in Ethiopia showed that HIV patients were more likely to have TB than HIV negative individuals[6]. The peak HIV prevalence is in the age group of 35–45 which is consistent with previous report[6]. HIV prevalence is higher among housewives compared to the others. Previous assessment showed that housewives are among the most at high risk groups for HIV infection as a result of the extramarital unprotected sexual affairs of their partners[29], [30].

Cough, fever, weight loss and night sweating do not predict the presence of active TB among TB suspects in this study. Recent reports showed that single symptoms of TB such as cough or fever are not sensitive and specific enough to predict the presence of TB. A combination of symptoms should be evaluated for the presence of TB[31], [32].

Knowledge of the TB suspects and a contact history were the two major risk factors for active TB in this study population. Individuals who had good knowledge about the transmission methods of TB might protect themselves from the diseases. Several previous reports have shown that contact history is a strong risk factors for the development of active TB[33], [34], [35]. Number of persons per room which is a proxy indicator of crowding does not predict active TB which is similar to a study conducted in southwest Ethiopia (unpublished data). Frequency and intimacy of contact may be important predictors than the crowding index. History of smoking does not have an association with active pulmonary TB which is in contrast to many literatures[36], [37] but in agreement with a study done in Ethiopia.

This study clearly indicated the importance of collaboration of the two diseases. In Africa where the prevalence of both diseases is high, it is quite important to consider both diseases in programming interventions and disease management. Separate planning of individual diseases will result a clear missed opportunities. In our study contact history is a predictor of active TB. This signifies the importance of contact tracing, to identify the contacts of index cases and possible treatment of latent TB cases.

Inclusion of all the health centers in Addis Ababa, use of primary data, and adequate sample size are some of the strength of this study. However, this study has some limitations. First, culture was not done which may introduce misclassification bias. Second, we couldn't follow the outcome of the non-TB patients. Third, we did not measure previous history of TB which might have implication for the finding.

In conclusion, the prevalence of TB among TB suspects is high. Fifty percent of the HIV positive TB suspects had TB. Knowledge and contact history with a TB patient in the family were strong predictors of TB. Case finding among TB suspects should be intensified particularly among those who have contact history with a TB patient in the family.
